# A Pilot Study of a Panel of Ocular Inflammation Biomarkers in Patients with Primary Sjögren’s Syndrome

**DOI:** 10.3390/cimb45040188

**Published:** 2023-04-01

**Authors:** Ana Boto de los Bueis, Miguel de la Fuente, Rafael Montejano-Milner, Almudena del Hierro Zarzuelo, Elena Vecino, Arantxa Acera

**Affiliations:** 1 Ophthalmology Service, Hospital Universitario La Paz, 28046 Madrid, Spain; anam.botodelos@salud.madrid.org (A.B.d.l.B.); adelhierro24@gmail.com (A.d.H.Z.); 2 Experimental Ophthalmo-Biology Group (GOBE), Department of Cell Biology and Histology, University of the Basque Country UPV/EHU, 48940 Leioa, Spain; migueldelafuentelopez13@gmail.com (M.d.l.F.); elena.vecino@ehu.es (E.V.); 3 Ophthalmology Service, Hospital Universitario Príncipe de Asturias, 28805 Alcala de Henares, Spain; rafael.montejano@salud.madrid.org; 4 Ikerbasque, Basque Foundation for Science, 48001 Bilbao, Spain

**Keywords:** Sjögren’s Syndrome, biomarker, S100A6, MMP-9, CST4, in vitro diagnosis, antibody microarray, rheumatoid arthritis

## Abstract

Ocular diseases have a strong impact on individuals, the effects of which extend from milder visual impairment to blindness. Due to this and to their prevalence, these conditions constitute important health, social and economic challenges. Thus, improvements in their early detection and diagnosis will help dampen the impact of these conditions, both on patients and on healthcare systems alike. In this sense, identifying tear biomarkers could establish better non-invasive approaches to diagnose these diseases and to monitor responses to therapy. With this in mind, we developed a solid phase capture assay, based on antibody microarrays, to quantify S100A6, MMP-9 and CST4 in human tear samples, and we used these arrays to study tear samples from healthy controls and patients with Sjögren’s Syndrome, at times concomitant with rheumatoid arthritis. Our results point out that the detection of S100A6 in tear samples seems to be positively correlated to rheumatoid arthritis, consistent with the systemic nature of this autoinflammatory pathology. Thus, we provide evidence that antibody microarrays may potentially help diagnose certain pathologies, possibly paving the way for significant improvements in the future care of these patients.

## 1. Introduction

Sjögren’s Syndrome (SS) is a chronic autoimmune disorder characterized by lymphocyte infiltration of exocrine gland tissue throughout the body, causing dryness mainly in the eyes and mouth, but often with additional arthralgias and damage to other body systems [1,2]. When the clinical manifestations that occur are associated with any other well-defined auto-immune disease, the disease is referred as secondary SS. Studies of the populations affected by SS are influenced markedly by the study design and also by the geographical area under study. The estimated prevalence of SS in the adult population in Spain, including both primary and secondary forms, is 0.33%, with a female-to male ratio of 6:1 and a mean age (standard deviation) of 57 (±13.4) years. Considering only primary SS, the estimated prevalence is 0.25% or 1 person in 400, which is comparable to European studies of a similar design [3]. Rheumatoid arthritis (RA) is the most prevalent autoimmune disease associated with SS in our area (20.3%) [4].

SS is a difficult to treat condition, and thus, it has an important impact on the patient’s quality of life (QoL), conditioning daily activities. Because the signs and symptoms of SS differ from person to person and can sometimes resemble those of other diseases, it can be challenging to identify. Some SS symptoms and signs can also be confused with side effects of various medicines [5]. The paucity of symptoms could delay a correct diagnosis and underestimate the grade of affectation. Although the initial clinical expression is usually a mild eye discomfort and/or fluctuating vision, SS may trigger corneal melt/perforation, uveitis, scleritis, retinal vasculitis, optic neuritis and consequently blindness [6]. Prompt recognition of such a potentially severe ocular surface inflammation will prevent ocular complications, increasing the ophthalmological explorations in these patients and treating the inflammation along with the DED.

The chronic ocular surface inflammation provoked by SS is due to direct damage of the corneal epithelium, whereas the associated dry eye disease (DED) is due to the functional impairment and destruction of the lacrimal glands. Aqueous tear deficiency causes tear film hyperosmolarity, which in turn activates epithelial inflammatory cascades [7]. The DED secondary to SS is less symptomatic but shows more severe medical signs and more blurred vision than the non-SS DED [8]. Ocular inflammation related to SS boosts the expression of some stress proteins at the ocular surface and in the tear fluid, which could serve as pathological markers. By definition, biomarkers are molecules through which a particular pathological or physiological process can be recognised or defined [9], favoring a better diagnosis and the better monitoring of diseases. A biomarker may be used to determine if a patient has a particular clinical condition, distinguish disease subtypes if relevant and indicate the best treatment, as well as permit better monitoring of the therapeutic responses and disease progression [10].

One of the main advantages of the human ocular system is that the corneal and conjunctival tissues, and their associated fluid (tears), are accessible for analysis. The identification of biomarkers, and the development of technologies to detect and quantify them with specific and sensitive tools, may be critical to understand disease pathophysiology and progression. Proteomic analysis revealed different signatures in tears and saliva compared to the serum of SS patients, with most of signatures being able to accurately distinct individuals suffering the disease from controls [11]; however, they demonstrated limited specificity for SS [12]. Thus, further exploration of potential correlations with clinical significance will by necessary to define best targets for novel treatments [11].

One of the best-studied biomarkers in ocular diseases is matrix metalloproteinase 9 (MMP-9). Ocular surface stress leads to a desquamation of the ocular surface epithelium, which is associated with a disruption of tight junctions at the apical face of the corneal epithelium. This phenomenon can be provoked by an uncontrolled increase in MMP-9 activity in the tear and this protease is associated with severe signs of inflammation. Therefore, MMP-9 is an interesting therapeutic target and pharmacological modifiers of MMP-9 expression have been seen to have differing degrees of success in modifying its activity in vivo [13,14,15,16,17,18,19,20]. MMP-9 is implicated in a range of pathologies, including DED [21], ocular surface injury [22], microbial keratitis [23], corneal ulceration [24], corneal neovascularization [25], keratoconus [26,27] and SS [28]. Pro-inflammatory mediators such as interleukin 1 (IL-1), tumor necrosis factor alpha (TNF-α), platelet-activating factor (PAF) and transforming growth factor-beta (TGF-β) are the main signaling proteins responsible for enhanced MMP-9 expression. Conversely, tissue inhibitors of metalloproteinases (TIMPs) hamper pro-MMP-9 conversion to active MMP-9, restoring homeostasis after stress. This equilibrium between pro-inflammatory and anti-inflammatory factors controls MMP-9 activity, although in pathological conditions an imbalance in ocular MMP-9 may be specifically triggered and it could serve as a diagnostic marker [29].

Calcyclin (S100A6) and cystatin S (CST4) have also been proposed as biomarkers that may be dysregulated in tear samples from individuals with an inflamed ocular surface [30]. S100A6 is a calcium-binding protein related to epithelial integrity and proliferation, and it is involved in the cellular response to different stress factors [31]. By contrast, CST4 is a protein with antimicrobial and defensive roles thanks to its cysteine protease inhibitor activity [32].

The discovery of biomarkers to better understand these clinical conditions and to evaluate new therapeutic strategies has improved diagnosis over recent years, as witnessed by the recently proposed diagnostic biomarkers and therapeutic targets [33]. Thus, the aim of the present study was to analyze the usefulness of an antibody microarray (AbMA) as a diagnostic and monitoring tool and the clinical relevance of biomarkers analyzed in the tear of patients with SS.

## 2. Materials and Methods

### 2.1. Tear Samples

We designed an observational, prospective, case-controlled study in which 32 subjects were enrolled, 22 with SS and 10 healthy individuals (CTs). In the SS group, 18.2% of the patients had RA, while the remaining patients had primary SS. This pathology diagnosis was based on the American College of Rheumatology Classification Criteria for Sjögren’s Syndrome [34], while RA was diagnosed by a rheumatologist.

This study was carried out by medically qualified personnel after approval of the institutional review board at the Hospital la Paz (Madrid, Spain: HULP code PI-2501), and in strict accordance with the tenets of the Helsinki Declaration regarding research in humans. Patients were recruited from the Ophthalmology Service Unit of the Hospital Universitario La Paz, and informed consent was obtained from all the participants after the nature and possible consequences of the study were explained to them. Tear samples were collected from the inferior lateral tear meniscus with no anesthesia, minimizing any irritation of the ocular surface or lid margin. The tear samples were obtained using Blaubrand microcapillary tubes from IntraMark (#7087-09, Wertheim, Germany), and they were then introduced into 0.5 mL Eppendorf tubes (#40420050, Hamburg, Germany) and stored at −80 °C until analysis. The patients included in this study suffered from tear deficiency due to SS. No clinical tests were performed on the day of tear collection to avoid altering the tear composition. Ophthalmological examination (slit lamp exploration and Schirmer’s test) and tear collection were performed separately on all subjects with an interval of at least 24 h to exclude possible infections, or any acute ocular surface complication, and to avoid altering the tear composition. Standard Schirmer’s tests with topical anesthesia were performed by placing a sterilized Schirmer-Plus Gecis strip (Neung sur Beuvron, France) in the lateral canthus, away from the cornea, and left in place for 5 min. Topical anesthesia was instilled about 3 min before performing the Schirmer’s test and the measurements were read in millimeters of wetting after 5 min. In addition, the SICCA OSS and an OSDI questionnaire were used to assess the symptoms of ocular irritation and their effect on vision-related function. The healthy volunteers were subjected to ocular surface examination to ensure that pathologies associated with the ocular surface were not present, and to confirm the absence of contact lens use, and allergic or atopic history as exclusion criteria.

### 2.2. Antibody Microarray Analysis of the Samples

**Figure 1:**
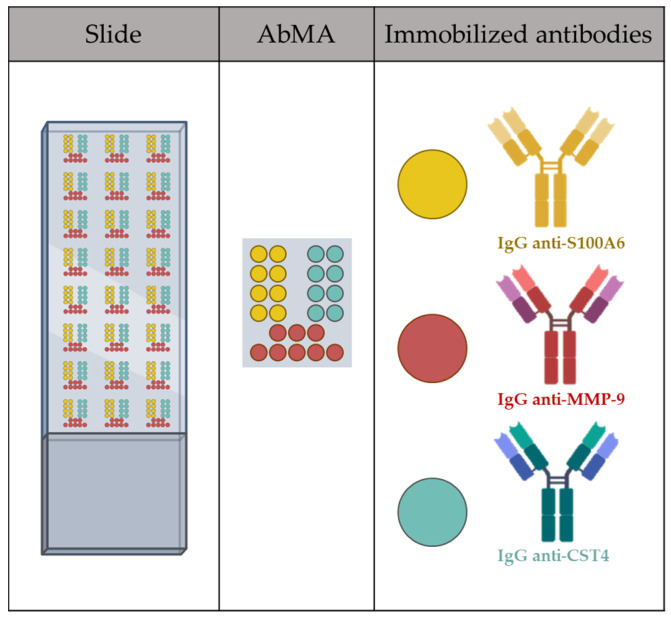
Representation of a microscope glass slide with the printed AbMAs. There were 24 AbMAs immobilized onto treated slides, each one with eight spots of the 3 biomarker IgGs at 200 µg/mL in SIVG (0.05%). Image created with BioRender.com.

Based on our previous research [27,35,36] and the use of previously characterized AbMAs [37,38], we analyzed the S100A6, MMP-9 and CST4 proteins in tear samples from a group of 32 individuals, control individuals (CTs) and SS patients. The use of AbMAs to analyze tear protein biomarkers was first validated by comparing MMP-9 tear biomarker quantification using an AbMA and ELISA [37]. The microarrays were fabricated on glass 76 × 26 mm microscope slides with 45° frosted ends (#1053057, LineaLAB, Badalona, Spain), preactivated with an acid treatment involving different washing steps to make the surface hydrophobic (EP2048534A4, IMG Pharma Biotech S.L., Derio, Spain). Each slide had 24 AbMAs printed on it in a 3 × 8 format (Figure 1), such that each AbMA had 8 replicate spots of IgG antibodies against the corresponding biomarkers: S100A6, CST4 and MMP-9 (respectively: #LAB769Hu71, #LAJ324Hu71 and #LAA553Hu71 from Cloud Clone Corp., Wuhan, China). The antibodies were immobilized at 200 µg/mL in SIVG printing solution (0.05%: IMG Pharma Biotech S.L., Derio, Spain) and one drop (30 nL) was printed at each spot using a non-contact microarrayer Nanoplotter (NP 2.1., GeSiM mbH, Radeberg, Germany). The AbMAs were printed on each slide at room temperature (RT) under controlled humidity (60%) and they were stored at −20 °C until use. For each batch printing, 4 glass slides of 24 immobilized AbMAs were prepared and 4 batch printings were carried out.

Three different antibodies, each directed against one of the selected tear protein biomarkers (S100A6, MMP-9 and CST4) were immobilized in the AbMAs and used to quantify the markers in the 32 tear samples. The immunodetection protocol was performed by first thawing and drying the slides for 30 min at RT in a drying chamber, and they were then washed three times for 5 min with phosphate buffer saline containing 0.01% Tween-20 (0.01% PBS-T) with agitation. The AbMAs were left in blocking solution (2.5% milk powder in 0.01% PBS-T) for 10 min at RT, washed with distilled water and the AbMAs were then incubated overnight at 4 °C in a humid chamber with the samples diluted 1:10 in 0.5% PBS-T and 0.01% sodium dodecyl sulfate (SDS: #436143, Sigma, St. Louis, MA, USA). Alternatively, the slides were probed with the biomarker standards (S100A6, #10939-HNAE; MMP-9, #10327-HNAH; and CST4, #11542-H08H; Sino Biological, Beijing, China) at the desired concentrations to establish the calibration curves. A final volume of 20 µL was used for each AbMA. After probing, the slides were washed twice with 0.5% PBS-T and once with 0.01% PBS-T, 10 min each with agitation. The AbMAs were probed for 1 h at RT in a humid chamber with the primary rabbit IgG antisera against human S100A6/MMP-9/CST4 (10 µg/mL, diluted in blocking solution). After incubation, the slides were washed once with 0.5% PBS-T and twice with 0.01% PBS-T for 5 min each with agitation. The slides were incubated for 1 h at RT in a humid chamber with an Alexa Fluor 647 conjugated secondary goat anti-rabbit IgG antibody (#ab150079: Abcam, Cambridge, UK) diluted 5 µg/mL in blocking solution. Subsequently, the slides were washed with agitation for 5 min each with 0.5% PBS-T, twice with 0.01% PBS-T, once with PBS and once with distilled water. The slides were then dried, and the fluorescent signal intensity of the spots was measured at 633 nm in an Agilent G2565BA Microarray Scanner (Agilent Technologies, Santa Clara, CA, USA), determining the protein concentration based on the standard curve intensities. For the microarray (a solid phase capture assay allowing simultaneous quantification of several biomarkers with replicates examined in the same sub-microarray, eight replicates per biomarker), 1 μL of each tear sample was needed. The protein concentration was determined based on the calibration curves established and using the ImageLab software (BioRad, Hercules, CA, USA).

### 2.3. Statistical Analysis

According to the small sample size, nonparametric tests were used for statistical comparisons. The Mann–Whitney U-test and Cliff’s delta value were calculated for assessing statistical differences of biomarkers presence between groups. The correlation with RA was obtained using the R studio software (Version 1.4.1106, Integrated Development for R, RStudio, PBC, Boston, MA, USA). Data were processed with GraphPad Prism 9.2.0 (GraphPad Software, San Diego, CA, USA). Statistical analyses were carried out with the SPSS 23.0 software (IBM, Armonk, NY, USA) and R studio software (Version 1.4.1106, Integrated Development for R, RStudio, PBC, Boston, MA, USA).

## 3. Results

### 3.1. Tear Samples

Tear samples were obtained from all the participants (CTs and SS patients) as indicated in the Materials and Methods, recording the sex and age of the individuals, and whether the SS patients also suffered from RA. In the Schirmer’s I tests a distance ≤5 mm in 5 min was considered abnormal and while all the CTs returned normal values, with a mean value of 11.5 mm, the mean Schirmer’s I test value for SS patients was 4.5 mm (Table 1). The Sjögren’s International Collaborative Clinical Alliance (SICCA) Ocular Staining Score (OSS) was also used to evaluate ocular inflammation. In this test, lissamine green (LG) dye was used to assess the conjunctiva and fluorescein dye to evaluate the cornea, giving equal numerical weight to corneal and conjunctival changes in accordance with their clinical relevance. Each stain was evaluated on a scale from 0 to 3 and an additional point was added if: (1) punctate epithelial erosions (PEE) with a diameter >4 mm were evident in the central cornea; (2) one or more filaments were seen anywhere on the cornea; or (3) one or more patches of confluent staining were found anywhere on the cornea. Thus, the maximum score possible for each cornea was 6 and the maximum possible score for each eye was 12 (the sum of the cornea and conjunctiva scores). Indeed, while a high score in corneal staining reflects a large number of PEE, a high conjunctival score reflects many dead cells [39]. The SICCA OSS values for the controls were 0, whereas the mean value for the SS patients was 5.9 (Table 1).

In addition, the ocular surface disease index (OSDI) questionnaire was used to assess the symptoms of ocular irritation and their effect on the individual’s vision [40], and they were classified as having a normal ocular surface (0–12 points), mild (13–22 points), moderate (23–32 points) or severe (33–100 points) ocular surface disease based on these OSDI scores. The mean OSDI in SS patients was 37.8 (severe ocular surface inflammation), while it was 2.4 for the CTs (normal ocular surface).

As a limitation of the study, it should be noted that the groups are not sex-matched. The SS group presents a majority of women (90.9%). However, this difference is partly due to the influence of sex on certain ocular pathologies, as in the case of SS, which, according to several studies, is more prevalent in women [41,42].

### 3.2. Antibody Microarray Analysis of Pathological Samples

**Figure 2:**
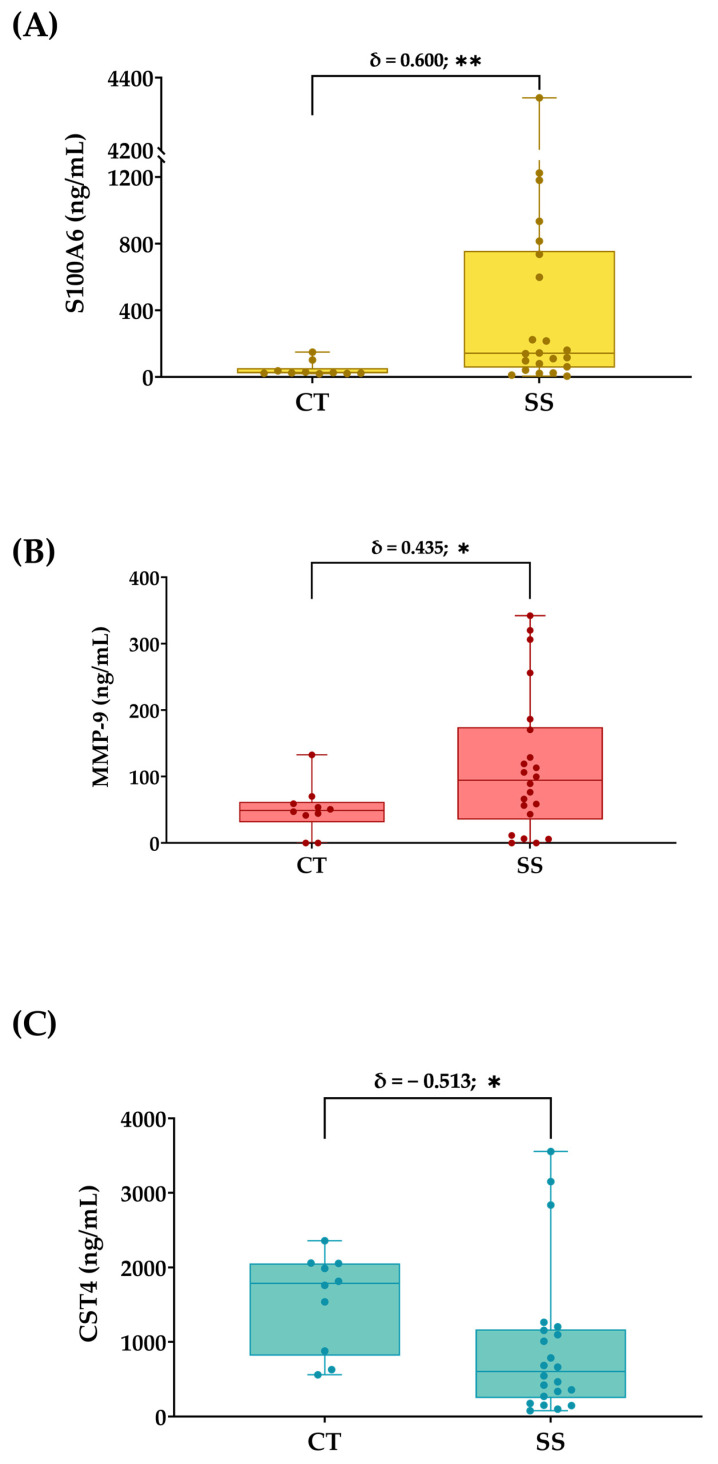
Tear S100A6 (**A**), MMP-9 (**B**) and CST4 (**C**) concentrations (ng/mL) in the groups of patients suffering SS relative to the healthy controls (CTs). Cliff’s delta values are displayed for each comparison. In addition, statistical significances are displayed: *, *p* < 0.05; **, *p* < 0.01.

The biomarkers S100A6, MMP-9 and CST4 were analyzed in 32 tear samples collected from CTs (*n* = 10) and SS patients (*n* = 22) using an AbMA (Table 1, Figure 2), and Cliff’s delta values were calculated to quantify the difference between the control and pathological groups. Significant differences in the concentration of the three biomarkers were seen when the two groups were compared, highlighting the relationship between the presence of the biomarkers and the individual’s physiopathology (presence of RA). Regarding S100A6, a Cliff’s delta value of δ = 0.600 was obtained by comparing the healthy CTs and the SS group. For MMP-9, the Cliff’s delta value was δ = 0.435 for the CTs versus the SS group. For CST4, this value was δ = −0.513 (Figure 2).

Cliff’s delta values over |0.474| reflect a large difference between the two groups and values in the interval |0.330|–|0.474| reflect a mild difference between the groups. Bearing this in mind, the S100A6 δ value (δ = 0.600) indicated a major difference between the CTs and SS patients. Likewise, a mild difference was evident when the δ value for MMP-9 between the SS patients and CTs was considered (δ = 0.435). By contrast, the CST4 δ value (δ = −0.513) indicates a major difference between the CTs and SS patients, reflecting a negative relationship.

When the concentration of each biomarker was assessed in the group of patients with SS, all showed statistically significant differences with respect to the CTs, with the changes indicating an upregulation of S100A6 and MMP-9, and a downregulation of CST4 in SS patients (Table 2). The most notable difference was found in the S100A6 biomarker in the SS patient’s tear, the concentration of which was significantly higher in SS patients (513.0 ± 941.7 ng/mL) than in the CTs (45.4 ± 43.9 ng/mL), a 10.31-fold increase (*p* = 0.0071). The increase in the MMP9 concentration was also significantly higher in the tear of the SS patients (116.6 ± 106.0 ng/mL) than in the tear of the CTs (47.3 ± 37.4 ng/mL, *p* = 0.045) and there was a significant decrease in the CST4 concentration in the SS group (930.3 ± 995.8 ng/mL) relative to the CTs (1383.2 ± 608.7 ng/mL, *p* = 0.0155). Despite the dispersion of the data and the relatively small sample number, the biomarker concentrations were significantly different in both study groups.

### 3.3. Relationship between Rheumatoid Arthritis and the Presence of the Biomarkers

**Figure 3:**
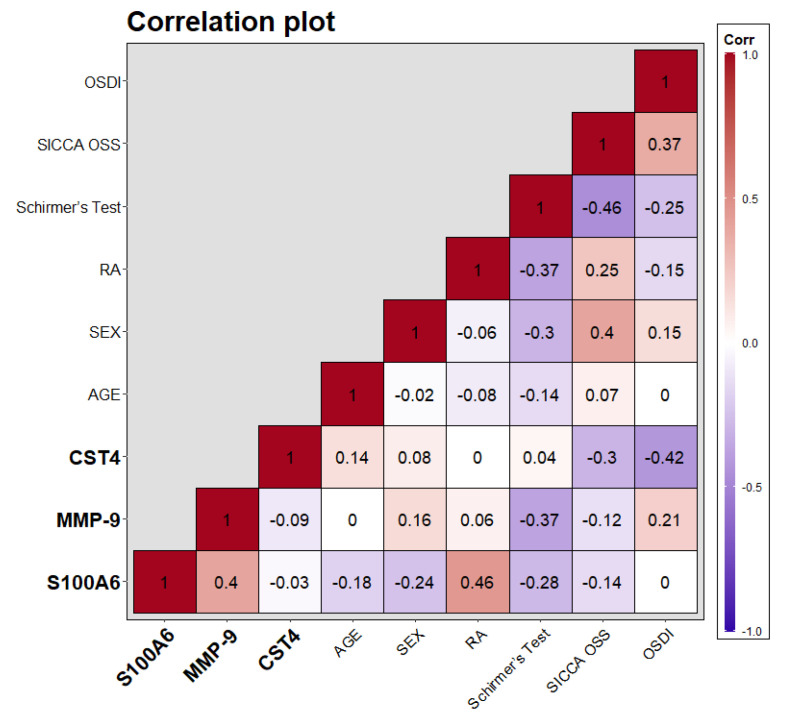
Correlation matrix of the S100A6, MMP-9 and CST4 biomarkers (in bold) in the tear samples and the clinical variables of all the individuals. The r value for each correlation is shown in the squares, and the color scale indicates the degree of significance and its direction (negative in blue, positive in red).

In order to assess the relationship between the concentrations of S100A6, MMP-9 and CST4 in tear samples and the individual’s pathological status, we tested the correlation between the presence of the biomarkers and RA (Figure 3). Only S100A6 was significantly correlated with RA, with an r value higher than |0.40| (r = +0.46). We calculated the *p*-value for the correlation between the presence of S100A6 in patients suffering RA and SS as opposed to its presence in patients not suffering RA, obtaining a coefficient of 0.008. That is, we can reject the null hypothesis (*p*-value = 0.008 < 0.01) and accept the alternative one, assuming that the concentration of S100A6 and the presence of RA-SS are significantly and linearly correlated. To calculate the significance of this relationship, a Pearson’s correlation was employed due to the need to compare continuous versus categorical variables.

## 4. Discussion

In SS, lymphocytes infiltrate exocrine glands throughout the body, particularly salivary and lacrimal ones, becoming a chronic autoimmune disorder [43]. Nowadays, the diagnosis is typically made using a combination of symptoms described by the patient, evaluations of dryness in the mouth and eyes, and blood tests to check for antinuclear antibodies. It can be challenging to diagnose and administer the proper therapy because many of these signs and symptoms are similar to those of other inflammatory diseases. In the current study, AbMAs developed previously [30,38], were used to measure the changes in CST4, S100A6 and MMP9 protein in the tears of patients with SS to assess if these biomarkers might serve to test the ocular involvement and active inflammation in this disease, and naturally, if they could be used to monitor the response to therapy. We choose these biomarkers based on our previous data and that described elsewhere [44]. CST4 is a natural cysteine protease inhibitor with antimicrobial activity. The lacrimal glands produce this extracellular tear protein, which is also found in the secretions of the meibomian glands. [45,46]. CST4 binds to bacterial lipopolysaccharides, suppresses endogenous, bacterial, and parasitic protozoa proteases, and appears to have some direct immunomodulatory effects. [47]. CST4 concentration in tears have been registered in patients with ADDE, blepharitis, Fusarium keratitis and MGD [48,49]. S100A6 is a protein, found in the tear fluid, involved in several processes, such as calcium-binding/epithelial integrity and growth [50], and it has been seen to be upregulated in SS [51]. MMP9 is an enzyme implicated in the matrix collagen IV and V digestion. MMP9 has been tested in candidate immunodetection and activity detection experiments, but it has not been identified in objective massive proteomics analyses of DED tears. Here, a batch of 32 tear samples from 22 SS patients and 10 CTs was analyzed to evaluate the diagnostic potential of this AbMA in a disease with a very limiting tear volume. The panel of biomarkers selected were able to confirm the pathophysiological status of each group defined through the Schirmer’s I test, SICCA OSS and OSDI. S100A6, MMP-9 and CST4 were initially quantified in all the tear samples using the AbMA developed and relative to the reference standards. To assess the diagnostic capacity of these analytes, tear biomarker concentrations were compared among the distinct sample types and the Cliff’s value was calculated as a useful complementary analysis to test the corresponding hypothesis [52]. In short, tear S100A6 and MMP-9 are elevated in SS patients, albeit to a different extent, which ratifies the diagnostic potential of these biomarkers as their presence is enhanced in conditions of ocular surface inflammation, as described previously [28,37,38]. Moreover, CST4 is downregulated in SS, again in concordance with earlier data [30,38].

We matched tear S100A6, MMP-9 and CST4 values with the presence of RA to study the diagnostic information provided by this panel of biomarkers. S100A6 is correlated with RA, the most common autoimmune condition that accompanies SS, with SS estimated to arise in 20% of patients with RA [53]. However, while SS and RA have distinct clinical and pathophysiological features [54], the majority of patients with early inflammatory polyarthritis have an undifferentiated disease, making RA diagnosis difficult at the beginning of the disease [55]. Some authors argue against the justification for using American College of Rheumatology (ACR) criteria or any criteria to define RA in patients with early inflammatory polyarthritis [56]. Traditional biomarkers for SS include SS-A/Ro, SS-B/La, antinuclear antibody (ANA) and rheumatoid factor (RF), although these are not specific to SS and they may also be detected in other autoimmune disorders [57,58]. Even with new blood diagnostic tools such as the Sjö^®^ Diagnostic Test (Bausch & Lomb, Rochester, NY, USA), the sensitivity and specificities of these biomarkers remain below 90% [53]. In terms of RA, serum RF can be detected in 70–80% of RA patients but it is not a very specific biomarker [59]. Antibodies against citrullinated peptides/proteins, carbamylated peptides or peptidylarginine deiminases (PADs) are sensitive and specific biomarkers that can precede the appearance of RA [60]. However, the diagnostic sensitivity of anti-CCP antibodies decrease to 70% in very early RA, and there is a relatively high frequency of anti-CCP2 antibodies in patients with non-RA connective tissue disorders, such as SLE (15%), SS (14%), polymyositis/dermatomyositis (23%) and scleroderma (16%) [61]. Our study is a preliminary analysis, but the results suggest that maybe S100A6 could be of some help to improve the detection of early RA in patients with primary SS.

These findings highlight the need for reliable objective measures and tools that can be used to diagnose RA. In this sense, there has been increasing interest in the study of biomarkers associated with neutrophils given their possible role in autoimmune diseases. Upon activation, neutrophils die in a process called NETosis in which chromatin is extruded to form a nuclear extracellular trap (NET), a structure that could serve as an autoantigen and that has been involved in the development of autoimmunity [62]. NETosis plays a fundamental role in the pathogenesis of RA and patients with RA have higher concentrations of circulating inflammatory cytokines, including TNF-α and IL-17A. Both recombinant TNF-α and IL-17A induce significant NETosis in RA neutrophils and hence, some S100 family proteins that are involved in regulating proliferation and inflammation may be elevated in RA patients [63]. In juvenile RA, levels of calprotectin and S100A12 are strongly influenced by disease activity and systemic therapy [64], and S100A12 proved to be the best single predictor of disease flare-up after the withdrawal of therapy [65].

Here we detected an increase in S100A6 expression in the tears of patients with RA-SS relative to the concentration of this protein in healthy subjects. S100A6 has been demonstrated to fulfill a role in other inflammatory autoimmune diseases such as primary biliary cholangitis and systemic sclerosis of the lung [66], consistent with our observations. Other than S100A6, S100A8/9/calprotectin has also been studied in inflammatory diseases, especially in RA, confirming the influence of this protein family in the pathology [67,68]. Monitoring S100A6 as a biomarker in tear samples from patients could be particularly useful since tear analysis is a non-invasive test, and as this technique requires only a call volume of tear, it is not bothersome to the patient. This study demonstrates that not only can tear S100A6 be quantified in human samples but that high concentrations of this biomarker could be related to RA. In any case, these results should be taken with caution since this is a pilot study involving only a few patients, and further multicenter studies will be necessary on a larger number of age-matched patients to confirm these relationships, a limitation of this study, it would also be appropriate to extend these studies to serum samples as well.

On the other hand, we showed a decrease in the concentrations of CST4 in the tear of patients with SS relative to that of the CTs. These findings corroborate previous studies in which lower concentrations of this biomarker were detected in the tear of individuals suffering different ocular inflammatory pathologies [30,44,48,49,69] and in saliva from patients with SS [70]. MMP-9 was the third biomarker analyzed and it was found at higher concentrations in SS patients than in healthy individuals. This is consistent with current data regarding tear MMP-9 overexpression in association with ocular inflammation [22,24,35,37,71].

This study represents a valuable proof-of-concept that demonstrates the diagnostic potential of analyzing biomarkers of inflammation in tear samples available in minimal or limiting volumes, offering the advantage of minimally invasive sampling. In terms of SS, about half of the patients are estimated to be undiagnosed [3]. Limited tear fluid is one of the most significant manifestations of SS, although this pathology should not be wrongly diagnosed as DED since this is simply a consequence of SS. It is fundamental to correctly detect SS in order to avoid treating only some of the symptoms, such as with the use of artificial tears for DED as opposed to targeting the cause of the disease. For instance, reducing and maintaining ocular surface inflammation at a minimum in SS may hinder or delay the progression of ocular complications, such as corneal ulcers and vision loss [72]. It is also important to identify RA in patients with SS in order to explore and treat the systemic manifestations of this disease. Furthermore, imprecise diagnosis or an excessive delay in reaching a correct diagnosis might affect the patient’s psychological well-being due to a deterioration in their QoL associated with ocular diseases [73,74].

Furthermore, it has been demonstrated that the performance of AbMA solid phase capture assays surpasses those of the traditional ELISA technique [75]. These characteristics make AbMAs one of the best alternatives to study biomarkers, particularly in tears, given the small sample volume required, the ease of detecting various biomarkers and the good sensitivity. These issues are key when analyzing tear fluid from patients with SS, as it can be very difficult to obtain a microliter of sample due to their symptoms of ocular inflammation and DED [1,6,53], making such samples scarce and valuable.

In summary, we demonstrate here that AbMA technology is useful to detect multiple biomarkers and that it can be used to analyze small volumes of tear fluid, a fundamental feature when assaying tear samples in patients with compromised tearing. S100A6 has been shown to play a crucial role in inflammation and here, elevated tear concentrations of this biomarker in SS patients appear to be associated with RA. Further research should be carried out to confirm this phenomenon and this study clearly has certain limitations related to the number of samples due to the difficulty of obtaining capillary tear fluid from patients with ocular inflammation (SS). It should also be taken into account, as a limitation of the study, the lack of a study group of RA patients without SS. Nevertheless, this is still a valuable proof-of-concept demonstrating the diagnostic potential of analyzing inflammatory biomarkers in tear samples with minimal and limiting volumes, not only to detect ocular diseases but also, to diagnose and monitor systemic pathologies, taking advantage of minimally invasive sampling.

## 5. Conclusions

In conclusion, we have developed an AbMA solid phase capture assay to detect S100A6, MMP-9 and CST4 in human tear samples. With this technology we were able to study a cohort of 32 samples from healthy controls (*n* = 10) and patients with SS (*n* = 22), revealing a positive relationship between the presence of S100A6 and RA. These data confirm the usefulness of this type of technology for diagnostic purposes and above all as a tool to be able to evaluate the results of treatment at the ocular level. The involvement of the S100A6 protein in inflammation of the ocular surface must be validated in a larger population of patients, which would pave the way to make advances in the treatment of these conditions, and to monitor patient responses using sensitive and specific tools such as AbMAs.

## Figures and Tables

**Table 1:** The demographics, clinical data and biomarker quantification for each group and the nature of each donor is indicated: Sjögren’s Syndrome (SS) or control (CT).

Variables, Units	SS	CT
*n*	22	10
Age, years	59.2 (13.4)	54.2 (10.5)
Sex (F/M), %	90.9/9.1	60.0/40.0
RA (Y/N), %	18.2/81.8	0.0/100.0
Schirmer’s Test, mm	4.5 (4.6)	11.5 (3.3)
SICCA OSS, score	5.9 (3.4)	0.0 (0.0)
OSDI, score	37.8 (33.2)	2.4 (2.9)
S100A6, ng/mL	513.0 (941.7)	45.4 (43.9)
MMP-9, ng/mL	116.6 (106.0)	47.3 (37.4)
CST4, ng/mL	930.3 (995.8)	1564.1 (645.3)

Note: The results are displayed as the means (standard deviation), except for the sex and presence of RA, which are shown as the percentage of females (F)/males (M) or yes (Y)/no (N), respectively: *n*, number of subjects.

**Table 2:** Dysregulated biomarkers comparing the SS versus CT values.

Variables	*p*-Value	Fold-Change
S100A6	0.0071	10.31
MMP-9	0.0462	1.46
CST4	0.0155	−0.41

*p* < 0.05 statistical significances; fold change >1.5 for upregulated proteins; fold change <0.5 for downregulated proteins.

## Data Availability

The data presented in this study are available on request from the corresponding author. The data are not publicly available due to patient anonymity.
